# The Impact of Contact Isolation on the Quality of Inpatient Hospital Care

**DOI:** 10.1371/journal.pone.0022190

**Published:** 2011-07-21

**Authors:** Daniel J. Morgan, Hannah R. Day, Anthony D. Harris, Jon P. Furuno, Eli N. Perencevich

**Affiliations:** 1 Department of Epidemiology and Public Health, University of Maryland, School of Medicine, Baltimore, Maryland, United States of America; 2 VA Maryland Health Care System, Baltimore, Maryland, United States of America; 3 University of Iowa, Carver College of Medicine and Iowa City VA, Iowa City, Iowa, United States of America; University of Hong Kong, Hong Kong

## Abstract

**Background:**

Contact Isolation is a common hospital infection prevention method that may improve infectious outcomes but may also hinder healthcare delivery.

**Methods:**

To evaluate the impact of Contact Isolation on compliance with individual and composite process of care quality measures, we formed four retrospective diagnosis-based cohorts from a 662-bed tertiary-care medical center. Each cohort contained patients evaluated for one of four Centers for Medicare and Medicaid Services (CMS) Hospital Compare process measures including Acute Myocardial Infarction (AMI), Congestive Heart Failure (CHF), Pneumonia (PNA) and Surgical Care Improvement Project (SCIP) from January 1, 2007 through May 30, 2009.

**Results:**

The 6716-admission cohort included 1259 with AMI, 834 with CHF, 1377 with PNA and 3246 in SCIP. Contact Isolation was associated with not meeting 4 of 23 individual hospital measures (4 of 10 measures were not met for care provided while patients are typically isolated). Contact Isolation was independently associated with lower compliance with the composite pneumonia process-of-care measure (OR 0.3, 95% CI 0.1–0.7). AMI, CHF and SCIP composite measures were not impacted by Contact Isolation.

**Conclusions:**

Contact Isolation was associated with lower adherence to some pneumonia quality of care process measures of care on inpatient wards but did not impact CHF, AMI or SCIP measures.

## Introduction

Contact Isolation is an intervention to prevent the spread of infectious diseases that is used on approximately one in five inpatients [1], [2]. Contact Isolation places patients in private rooms and requires that hospital staff don gowns and gloves prior to entering patient rooms [2]. Healthcare workers visit patients on isolation approximately half as frequently as non-isolated patientS [1], [3]–[5]. While in the past Contact Isolation was primarily used in intensive care unit (ICU) patients, it is now increasingly used in non-ICU patients as a critical element in the nationwide Department of Veterans Affairs methicillin-resistant *Staphylococcus aureus* (MRSA) prevention initiative as well as legislated active surveillance programs for MRSA in Illinois and other US states [6]–[7]

Process-of-care quality measures for acute myocardial infarction (AMI), congestive heart failure (CHF) pneumonia (PNA) and Surgical Care Improvement Project (SCIP) are advocated by the Centers for Medicare and Medicaid Services (CMS), The Joint Commission (TJC) and the National Quality Forum [8]–[12]. They are publicly reported through the Hospital Compare website (http://www.hospitalcompare.hhs.gov/) as a means by which payers, providers and the general public can evaluate hospitals, with the intention of improving the quality of care provided (a description of each individual measure can be found in Appendix S1). Adherence to publicly reported process-of-care quality measures has increased over time, both through better documentation and improved performance [10].

In a frequently cited paper from 2003, a retrospective cohort study found multiple indicators of lower quality of care among isolated patients, however standardized process-of-care quality measures were not broadly assessed [13]. Investigators evaluated specific measures of CHF and AMI treatment. These measures appeared to be unaffected by Contact Isolation if the task they measured was performed in the emergency room where isolation is generally not used. However, in isolated patients, CHF and AMI care performed after admission was generally worse. Furthermore, general indications of quality were worse in isolated patients, including incorrect vital sign documentation and missing nursing and physician notes [13]. Recent guidelines for infection prevention have highlighted the need to prevent worse care associated with Contact Isolation [2], [14].

To examine the association between exposure to Contact Isolation and adherence to process-of-care quality measures collected by hospitals throughout the US we evaluated quality in four retrospective diagnosis-based cohorts of inpatients adjusting for comorbidities and severity of illness.

## Methods

This study was conducted at the University of Maryland Medical Center (UMMC), a 662-bed tertiary acute care teaching hospital with 170 adult intensive care unit beds in Baltimore, MD. Clinical data were collected on patients admitted during the 28-month period, January 1, 2007 through May 30, 2009, a period when all measures were being collected. All data were collected in the process of routine clinical care, patient billing and public reporting (in the case of process of care measures). All patients were ≥18 years of age and admitted for no longer than 120 days. Within each database, when a patient had multiple admissions, data were only analyzed for the first admission to eliminate non-independence. This study was approved by the University of Maryland Institutional Review Board (IRB). A waiver of informed consent was approved by the IRB given all data was historical in nature.

### Databases

Using the national standard, process of care measures were collected by a hospital quality improvement nurse in accordance with guidelines from the Centers for Medicare and Medicaid Services and The Joint Commission. This quality improvement nurse was not aware of the study hypothesis or the patient isolation status. Quarterly audits were conducted by CMS with greater than 80% agreement throughout the study period. We created four retrospective cohorts, using all admissions to UMMC with a primary diagnosis of acute myocardial infarction (AMI), congestive heart failure (CHF) and pneumonia (PN). These were selected as the standard measures collected and reported by Hospital Compare. For surgical care improvement project (SCIP) measures, the following operations were reviewed: all cardiac surgery (including coronary artery bypass grafts), knee and hip replacement surgery, vascular surgery, colon surgery and hysterectomy. In addition, 25% of other major surgeries selected at random were included for SCIP measures (selected from over 300 operations identified by CMS and TJC). Patients with appropriate diagnoses were evaluated for measures according to CMS/TJC guidelines.

Measures of care generally occurring at time of admission in the emergency room or operative room are: CHF2, CHF3, AMI1, AMI3, AMI6, PNA1, PNA6B, SCIP1, SCIP2, SCIP6, SCIP7, SCIP VTE1 & SCIP VTE2. Measures of care generally occurring in the ICU are: SCIP4. Measures of care generally occurring on hospital floors include: CHF1, CHF4, AMI2, AMI4, AMI5, PNA2, PNA4, PNA7, SCIP3. (see Appendix S1 for a description of each measure)

Patient demographic, admission and discharge information were obtained from the UMMC Central Data Repository (CDR) that contains demographic, billing, pharmacy and laboratory information. CDR data used for this study has been validated as 99% accurate compared to chart review [15]. Contact Isolation was defined as the presence of an indicator for multidrug-resistant (MDR) bacteria isolated from clinical or surveillance cultures. MDR bacteria include methicillin-resistant *Staphylococcus aureus* (MRSA), vancomycin-resistant enterococcus (VRE) and gram-negative bacteria (e.g. *Pseudomonas aeruginosa*, *Acinetobacter baumannii*) susceptible to ≤two antibiotic classes not including polymyxin or tigecycline [2]. Contact Isolation is applied to patients with an indicator in the electronic medical record upon admission to the hospital. Patients who were found to have an MDR bacteria during a previous admission or who are noted to have an MDR bacteria at time of transfer are isolated at time of admission. Patients isolated for a culture result during the current admission are isolated within 24 hours of positive result. Within this hospital, active surveillance culturing is performed for MRSA and VRE on all intensive care unit patients and MRSA surveillance for general patients who answered “yes” to either of the questions “have you been admitted to the hospital in the last year?” or “do you have a wound?” Active surveillance culturing is performed for MDR gram-negative bacteria in patients transferred from other hospitals [16]. During the study period, universal Contact Isolation was used within the medical intensive care unit and consists of gown and gloves for all patient contact, regardless of presence of MDR bacteria.

### Validation

The electronic indicator for Contact Precautions was validated in a random sample of admissions and was found to be 96% sensitive compared to paper records (77/80).

Severity of illness was measured using the 3M Grouper all-payer-refined mortality risk (3M, Maplewood, MN) conducted by financial services as a part of routine billing. 3M Grouper codes represent severity of illness for a given diagnosis and are scored from 1–4 [17]. Comorbidities were measured using the Charlson Comorbidity Score [18].

### Analysis

Each cohort was formed based on one of four possible primary diagnoses (e.g. AMI or PN) during the index admission to our hospital during the study period. Each individual admission was the unit of analysis. For patients with multiple admissions only the first admission during the study period was analyzed to correct for lack of independence for the same patient.

Individual process of care measures within a diagnosis were combined to form a composite measure as proposed by Nolan & Berwick and used by others [19], [20]. Briefly, composite items were selected based on previous literature reports combined with number of total admissions available given different combinations of measures. To qualify as adherent to the composite measure the patient admission must have been shown to receive all individual sub-measures. Given the inability to develop a composite score for pneumonia with three variables that would include >100 patients, a composite measure was created including patients who had at least 2 of 3 measures of inpatient care evaluated (PNA 2, 4 or 7) [19]. One measure common to three of the four cohorts was smoking cessation advice and counseling. Because this measure was identical in each cohort, a new cohort was formed examining all patients who qualified for this measure, regardless of diagnosis (from cohorts AMI, CHF and PNA). Each patient was evaluated once, on their first visit, even if they had multiple admissions for different diagnoses.

The primary outcome of interest was 100% adherence to the composite measure for each cohort (AMI, SCIP, CHF and PNA). Bivariable analysis of predictor variables was conducted followed by multivariable logistic regression. Bivariable analysis utilized chi-square or Fishers exact test for binary variables and t-tests or Wilcoxon-rank sum test for continuous variables, as appropriate. Those variables found to have a p-value<0.10 as well as the prespecified independent variable Contact Isolation were evaluated in the multivariable model. Length of stay and all-payer refined mortality risk were non-normally distributed and were dichotomized about the median. Collinearity was assessed with Pearson's or Spearman's rank test. Effect modification was assessed with stratified analysis. We report adjusted odds ratios (ORs) and 95% confidence intervals (CIs) from the multivariate logistic regression model.

Secondary analysis of each individual process of care measure was conducted using Chi-square or Fishers exact testing examining the impact of Contact Isolation on successful adherence to a measure on bivariable analysis. The Bonferroni adjustment was made for significance testing in secondary analysis to correct for multiple secondary outcomes (p value of <0.01).

All analyses were performed using Statistical Analysis Software (SAS), version 9.2 (SAS Corporation, Cary, NC).

## Results

A total of 7463 admissions had quality measures evaluated. Of these 6716 were first admissions for an individual patient within each dataset. Number of admissions per process-of-care-measure and basic demographic information for each measure are presented in Table 1. Bivariable analysis of exposures predicting each composite measure is presented in Table 2.

**Table 1:** Basic admission characteristics for all patients included in each Center for Medicare & Medicaid (CMS) process of care measure.

	Acute Myocardial Infarction	(AMI) (n = 1259)	Congestive Heart Failure (CHF) (n = 834)	Pneumonia (PNA) (n = 1377)	Surgical Care Improvement Project (SCIP) (n = 3246)
Age (median, interquartile ratio)	63 (53, 73)		59 (51, 69)	52 (43, 64)	58 (47, 69)
Gender (% female)	37.8%		40.8%	47.5%	44.2%
Isolated during admission	9.5%		19.2%	38.2%	12.6%
Charleson Comorbidity Index (median, quartiles)	2 (1,3)		3 (2, 4)	2 (1,4)	1 (0, 2)
Length of stay (median, quartiles)	3.0 (1.9, 6.7)		3.8 (2.1, 6.9)	4.0 (2.1, 9.3)	6.5 (4.3,11.2)
Admitted to Intensive Care Unit (ICU)	72.1%		29.5%	35.4%	57.3%
Admitted to same hospital in the year prior to index admission	7.1%		38.0%	49.5%	22.1%
All-payer-refined mortality risk*	Minor	18.0%	19.8%	40.7%	28.6%
	Moderate	30.1%	2.8%	3.4%	10.0%
	Major	28.2%	26.5%	22.9%	23.2%
	Extreme	23.7%	50.9%	33.1%	38.1%

*3M All-payer-refined mortality risk for each specific diagnosis [17].

**Table 2:** Bivariable analysis for each independent variable and the outcome of 100% adherence with each CMS process-of-care composite measure comparing patients *without* 100% adherence with composite measures to those patients *with* 100% adherence to composite measures [19], [20].

	AMI	CHF	PNA	SCIP
	(2,4,5)	(1–3)	(2 of 3: 2,4,7)	(1–3,6)
**Age (mean)**	62.7 vs. 57.0	56.9 vs. 56.4	74.0 vs. 66.0**	60.9 vs. 61.4
**Charlson Comorbidity Index (mean)**	2.4 vs. 2.2	2.8 vs. 3.1	2.1 vs. 2.6	1.9 vs. 1.5**
**Length of Stay in days (mean)**	11.5 vs. 5.0**	4.0 vs. 5.2	11.5 vs. 7.0*	8.9 vs. 7.5**
**All-payer-refined mortality risk (mean)**	2.7 vs. 2.4	2.8 vs. 2.7	3.6 vs. 3.0**	2.8 vs. 2.8
**Sex (% female)**	25% vs. 34%	30% vs. 34%	48% vs. 48%	46% vs. 44%
**Admitted to hospital in the past year**	18.8% vs. 7.0%^x^	31.3% vs. 43.3%^x^	39.1% vs. 31.7%	20.8% vs. 17.6%
**ICU during admission (mostly better in ICU)**	50.0% vs. 73.8%*	19.4% vs. 27.8%	69.6% vs. 34.2%**	52.5% vs. 66.9%**

x p<0.10.

*p<0.05,

**p≤0.01.

### Congestive Heart Failure (CHF) Cohort

834 patient-admissions were analyzed based on hospital admission with a primary diagnosis of CHF. Model building for predictors of adherence with the composite measure of CHF 1–3 identified admission in the past year as trending towards statistical significance on multivariable logistic regression (OR 0.6, 95% CI 0.3–1.0). Contact Isolation was not associated with worse CHF process-of-care quality composite measure (OR 1.0, 95% CI 0.5–2.2). (Table 3) No individual CHF CMS process-of-care quality measures were statistically associated with exposure to Contact Isolation. (Table 4)

**Table 3:** Logistic regression models for each composite outcome measure.

Outcome (100% adherence with composite measures)	Variable	Adjusted Odds Ratio and 95% Confidence intervals
**AMI composite**	Contact isolation	0.7 (0.1–5.0)
	Admission to ICU	2.5 (0.9–10.0)
**SCIP composite**	Contact isolation	0.8 (0.5–1.3)
	LOS>6.1 days	0.7 (0.5–0.9)
	Admission to ICU	2.0 (1.7–2.5)
**CHF composite**	Contact isolation	1.0 (0.5, 2.2)
	Admission in the year prior	0.6 (0.3–1.0)
**PNA composite**	Contact isolation	0.3 (0.1–0.7)
	Age>68 years	0.9 (0.8–1.0)

Those variables with an odds ratio <1 indicate an association between that variable and unsuccessful composite measure outcome. Each model was calculated independently forcing the variable contact isolation and including other variables that improved the descriptive ability of each model.

**Table 4:** Secondary analysis of individual process measures presented as proportion of isolated and un-isolated patients successful on each measure.

	Isolated	Not Isolated	Significance* (p value)
**Congestive Heart Failure (CHF)**			
CHF1 (discharge instructions) n = 692	88.5%	89.3%	0.96
CHF2 (Left ventricular function assessment) n = 755	99.3%	99.0%	0.77
CHF3 (ace/arb for left ventricular systolic dysfunction) n = 402	91.5%	91.3%	0.60
CHF4 (smoking cessation advice/counseling) n = 236	97.9%	97.9%	0.78
Composite CHF 1–3 n = 380	80.4%	82.7%	0.71
**Acute Myocardial Infarction (AMI)**			
AMI 1 (aspirin at arrival) n = 151	100%	99.3%	0.60
AMI 2 (aspirin at discharge) n = 1027	100%	98.8%	0.28
AMI 3 (ace/arb for patients with left ventricular systolic dysfunction) n = 205	95.2%	94.6%	0.89
AMI 4 (smoking cessation advice/counseling) n = 485	93.8%	98.7%	0.09
AMI 5 (beta-blocker at discharge) n = 1044	100%	98.1%	0.20
AMI 6 (beta-blocker at arrival) n = 122	100%	98.2%	0.72
AMI 7 excluded—thrombolytics not used in facility			
AMI 8 excluded—PCI (<10 per year in each group)			
Composite AMI 2,4,5 n = 441	96.6%	96.4%	0.39
**Pneumonia (PNA)**			
PNA1 (Oxygenation assessment) n = 613	100%	99.3%	0.19
PNA 2 (pneumococcal vaccine) n = 231	70.6%	83.6%	0.02
PNA 4 (smoking cessation advice/counseling) n = 478	90.7%	96.2%	0.02
PNA5 excluded—antibiotic timing (<10 per year in each group)			
PNA 6A excluded—antibiotic selection in ICU patients (<10 per year in each group)			
PNA 6B (antibiotic selection in non-ICU patients) n = 238	90.3%	95.7%	0.29
PNA 7 (influenza vaccination) n = 270	76.2%	86.1%	0.04
Composite of at least 2 of PNA 2,4 or 7 n = 187	78.6%	93.2%	<0.01
**Surgical Care Improvement Project (SCIP)**			
SCIP 1(prophylactic antibiotic received within 1 h prior to surgical incision) n = 1651	94.2%	94.2%	0.95
SCIP 2 (prophylactic antibiotic selection for surgical patients) n = 1749	97.1%	97.8%	0.33
SCIP 3 (prophylactic antibiotics discontinued within 24 h after surgery end time) n = 1564	84.3%	90.3%	0.05
SCIP 4 (cardiac surgery patients with controlled 6 am postoperative blood glucose) n = 1148	89.2%	92.6%	0.24
SCIP 6 (surgery patients with appropriate hair removal) n = 3003	97.2%	98.2%	0.19
SCIP 7(colorectal surgery patients with immediate postoperative normothermia) n = 248	62.5%	72.7%	0.34
SCIP VTE 1 (recommended VTE prophylaxis ordered) n = 994	93.8%	95.5%	0.42
SCIP VTE 2 (VTE prophylaxis received 24 hours prior/post surgery n = 992	88.4%	91.4%	0.28
Composite SCIP 1–3,6 n = 1563	79.6%	83.3%	0.21
Smoking cessation advice and counseling cohort (CHF, AMI & PNA4) n = 1199	92.1%	97.6%	<0.01

*Using the Bonferroni adjustment for multiple secondary outcomes, significance indicated by p value<0.01.

### Acute Myocardial Infarction (AMI) Cohort

1259 patient-admissions were analyzed based on hospital admission with primary diagnosis of AMI. Model building for predictors of adherence with the composite measure of AMI 2,4,5 identified a trend towards ICU stay as associated with better compliance on multivariable logistic regression (OR 2.5, 95% CI 0.9–10.0). Contact Isolation was not associated with worse AMI process-of-care quality composite measure (OR 0.7, 95% CI 0.1–5.0). (Table 3) No individual AMI CMS process-of-care quality measures were statistically associated with exposure to Contact Isolation. (Table 4)

### Pneumonia Cohort

1377 patient-admissions were analyzed based on hospital admission with primary diagnosis of pneumonia. The composite pneumonia process-of-care measure was significantly more likely to be missed in patients on Contact Isolation (6.8% vs. 21.4%, OR 0.3, p<0.01). Logistic regression analysis with adherence to the composite measure of pneumonia (PNA 2, 4 or 7) identified increasing age above 65 years of age (OR 0.9 per year, 95% CI 0.8–1.0) and Contact Isolation (OR 0.3, 95% CI 0.1–0.7) as associated with not reaching the pneumonia process-of-care quality composite measure. (Table 3) Three individual CMS process-of-care quality measures were less likely to be met in patients on Contact Isolation. After Bonferroni adjustment, statistical trends were noted for patients on Contact Isolation being less likely to meet measures PNA2 (pneumococcal vaccine prior to discharge for those >65 years of age; 16.4% vs. 29.4%, OR 0.5, p = 0.02), PNA4 (smoking cessation advice/counseling; 3.8% vs. 9.3%, OR 0.4, p = 0.02), and PNA7 (influenza vaccination; 13.9% vs. 23.8%, OR 0.5, p = 0.04). (Table 4)

### Surgical Care Improvement Project (SCIP) Cohort

3246 patient-admissions were analyzed based on hospital admission in which they underwent a surgical procedure included for SCIP. Predictors of adherence with the composite measure of SCIP 1–3 and 6 included admission to an ICU (OR 2.0, 95% CI 1.7–2.5) whereas length of stay longer than the median of 6.1 days (OR 0.7, 95% CI 0.5–0.9) was associated with worse adherence to the composite measure. Contact Isolation was not statistically significantly associated with worse SCIP process-of-care quality composite measure (OR 0.8, 95% CI 0.5–1.3). (Table 3) No individual SCIP CMS process-of-care quality measures were statistically associated with exposure to Contact Isolation. (Table 4)

### Smoking cessation advice and counseling cohort

The combined smoking cessation advice and counseling measure (from CHF, AMI and PNA cohorts, n = 1199) was significantly less frequently achieved in patients on Contact Isolation (7.9% missed measure vs. 2.4% missed measure, OR 0.3, p<0.01). (Table 3)

## Discussion

Most process-of-care quality measures are collected at hospital admission either in the emergency department or the operating room prior to exposure to Contact Isolation. None of the measures that are collected around the time of admission appeared impacted by exposure to Contact Isolation. However, the primary outcome of pneumonia care and smoking cessation advice and counseling were less likely to be achieved if a patient was on Contact Isolation. These elements of care generally occur while patients are on inpatient wards where Contact Isolation is more consistently used. Thus, our findings suggest that Contact Isolation, and not the underlying comorbidity associated with multidrug-resistant organism colonization, hinders delivery of care. If the organism colonization or the associated comorbidity hindered care, we would expect reduced compliance with process of care quality measures consistently throughout the patient admission.

Predictors of complete adherence with process-of-care composite measure differed by measure but were generally worse in patients with markers of chronic or severe illness such as having been admitted in the year prior to admission. Composite process-of care measures in AMI and SCIP were better with admission to specialized ICUs. Better AMI and SCIP process-of-care measures in patients admitted to an ICU was not expected. This may be explained by the fact that, in our hospital, there are ICUs dedicated to cardiac care and surgical care and both AMI and SCIP measures relate to processes that can be protocol driven. Patients with AMI who are admitted to a general ward may be on one of many wards, while those with a clear AMI syndrome are generally transferred from an outside hospital directly to the cardiac catheterization laboratory or the cardiac ICU where patient care managers round with the physician team, incorporating aspects of standard AMI quality measures. Patients with SCIP measures going to the surgical ICU also fit into protocols which likely improved adherence with measures.

The association between increased severity of illness (as measured by all-payer-refined mortality risk score, admission in the prior year or longer length of stay) and worse adherence to quality measures is not surprising given these patients are potentially sicker and therefore less likely to be evaluated and treated in a standard fashion [21]. Medically complex patients have been associated with better process-of-care measures; however these studies were over many types of hospitals and not exclusively in a tertiary care center, where the average level of complexity tends to be higher [22], [23].

Contact Isolation was negatively associated with process-of-care measures for pneumonia care and smoking cessation. Less documentation of smoking cessation advice and counseling was primarily identified in patients with myocardial infarction and pneumonia (AMI4, PNA4) and pneumococcal or influenza vaccination prior to discharge in patients with pneumonia (PNA2, PNA7). Contact Isolation may be a marker of more medically complex patients [24]. However, patients on Contact Isolation also have a potential barrier to care. Because of the time required to don gowns and gloves as well as typically being in a private room, isolated patients have approximately half as many health care worker visits as non-isolated patients [1], [3]–[5]. Patients on Contact Isolation also have longer admissions (in part because of difficulty obtaining long-term care facility placement), which could affect the process related to delivering instruction on smoking cessation and vaccinations [1], [13], [25]. The clinical impact of worse performance on these measures may be significant. Smoking cessation education has recently been questioned as a process-of-care quality measure because of difficulty in accurately measuring true delivery of cessation education [10]. However, increasing a patient's association between smoking and recent illness event may increase motivation to stop smoking. Vaccinations against Pneumococcus and influenza can improve outcomes in high risk patients [26], [27].

Those who provide care to hospital in-patients should be aware of potential negative associations with Contact Isolation. Hospital administrators and others involved in quality improvement should consider approaches to improving measures that appear to be affected by Contact Isolation. Future studies are needed to evaluate the impact of Contact Isolation on quality of care across multiple hospitals. These estimates need to be included in comparative effectiveness evaluation of infection prevention interventions that utilize Contact Isolation (e.g. MRSA active detection and isolation). In addition, as quality measures are rapidly changing, future measures that focus on care provided to inpatients should be evaluated for interactions with isolation or other concurrent performance improvement practices.

Our study has several potential limitations. These include being conducted at a single center, which may limit generalizability, as well as using retrospective administrative data. Only one primary outcome was statistically significant (pneumonia care) and differences in individual process measures were secondary outcomes. During the study period the MICU was using Contact Isolation for all patients, without regard to MDR bacteria status. In our analysis those patients without MDR bacteria in the MICU (but not the other 11 ICUs) were treated as not exposed which may have biased our findings towards the null.

As we continue to move towards greater accountability for process measures and greater use of interventions such as Contact Isolation as a means to prevent healthcare-associated infections, we must look carefully for unintended consequences of policy changes [10]. During the past five years, compliance with quality measures has dramatically increased with some evidence of correlation with lower mortality [10], [28], [29]. Hospital-associated infections have also decreased dramatically with widespread acceptance of the preventability of many infections [30], [31]. In order to maintain gains in overall quality of care delivered to an older and increasingly complex population of hospital inpatients, careful attention to unintended consequences of such interventions must be maintained. Interventions could be developed and tested to improve delivery of care for patients in Contact Isolation that retain the benefits of the intervention in preventing transmission of hospital-associated pathogens.

## Supporting Information

Appendix S1 Center for Medicare and Medicaid Studies (CMS) process-of-care measures collected during the study period.
(DOC)
