# Supplementary material for: The Impact of Contact Isolation on the Quality of Inpatient Hospital Care
Source: PLoS One. 2011 Jul 21;6(7):e22190. doi: 10.1371/journal.pone.0022190 (PMC3141007; doi:10.1371/journal.pone.0022190)
Supplement: Appendix S1 — Center for Medicare and Medicaid Studies (CMS) process-of-care measures collected during the study period. (DOC) [file pone.0022190.s001.doc]

Appendix 1: Center for Medicare and Medicaid Studies (CMS) process-of-care measures collected during the study period.

| **Congestive Heart Failure (CHF)** |
| --- |
| CHF1 discharge instructions |
| CHF2 Evaluation of left ventricular function |
| CHF3 angiotensin converting enzyme (ACE) inhibitor or angiotensin receptor blocker (ARB) for patients with left ventricular systolic dysfunction |
| CHF4 smoking cessation advice/counseling |
|  |
| **Acute Myocardial Infarction (AMI)** |
| AMI 1 aspirin at arrival |
| AMI 2 aspirin at discharge |
| AMI 3 angiotensin converting enzyme (ACE) inhibitor or angiotensin receptor blocker (ARB) for patients with left ventricular systolic dysfunction |
| AMI 4 smoking cessation advice/counseling |
| AMI 5 beta-blocker at discharge |
| AMI 6 beta-blocker at arrival |
| AMI 7 fibrinolytic medication within 30 minutes of arrival |
| AMI 8 percutaneous coronary intervention (PCI) received within 90 minutes of hospital arrival |
|  |
| **Pneumonia (PNA)** |
| PNA1 Oxygenation assessment |
| PNA 2 pneumococcal vaccine |
| PNA 4 smoking cessation advice/counseling |
| PNA5 initial antibiotic timing |
| PNA 6A appropriate antibiotic selection in ICU patients |
| PNA 6B appropriate antibiotic selection in non-ICU patients |
| PNA 7 influenza vaccination |
|  |
| **Surgical Care Improvement Project (SCIP)** |
| SCIP 1 prophylactic antibiotic received within 1 hour prior to surgical incision |
| SCIP 2 prophylactic antibiotic selection for surgical patients |
| SCIP 3 prophylactic antibiotics discontinued within 24 hours after surgery end time |
| SCIP 4 cardiac surgery patients with controlled 6 a.m. postoperative blood glucose |
| SCIP 6 surgery patients with appropriate hair removal |
| SCIP 7 colorectal surgery patients with immediate postoperative normothermia |
| SCIP VTE 1 recommended venous thromboembolism prophylaxis ordered |
| SCIP VTE 2 venous thromboembolism prophylaxis received 24 hours prior/post surgery |
|  |
